# Supplementary material for: AIBP controls TLR4 inflammarafts and mitochondrial dysfunction in a mouse model of Alzheimer’s disease
Source: J Neuroinflammation. 2024 Sep 28;21:245. doi: 10.1186/s12974-024-03214-4 (PMC11439205; doi:10.1186/s12974-024-03214-4)
Supplement: Supplementary file 1 — Supplementary Material 1 [file 12974_2024_3214_MOESM1_ESM.pdf]

Kim et al. **AIBP controls TLR4 inflammarafts and mitochondrial dysfunction in a mouse model of Alzheimer's disease**

Supplementary Figures

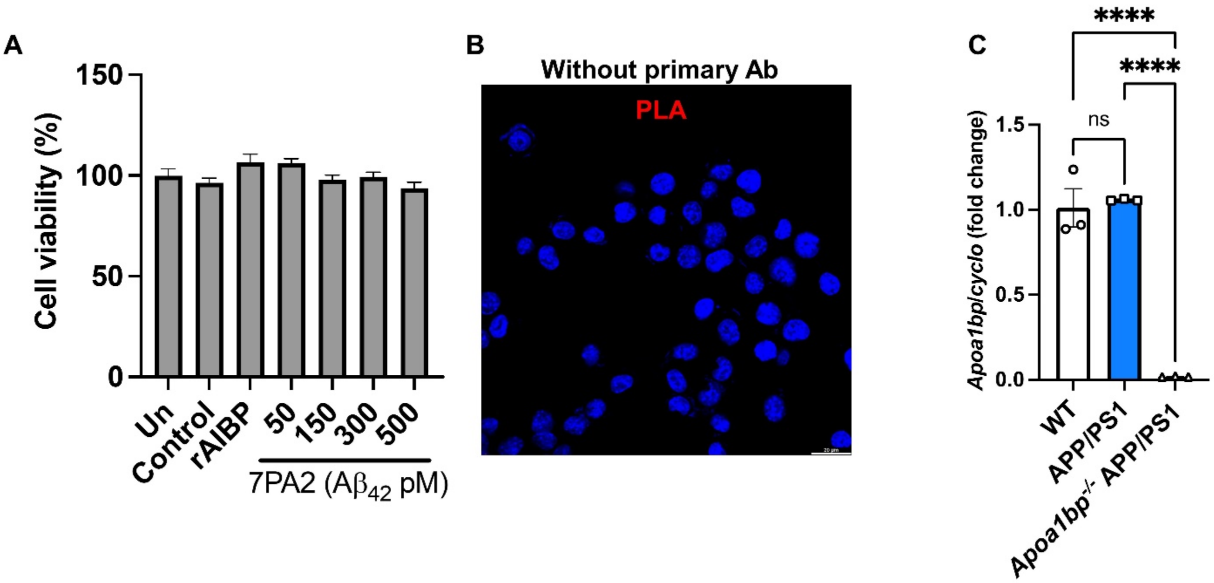

Figure S1. Controls for Figures 1 and 2.

(**A**) Cell viability of BV-2 cells incubated with non-conditioned media (Un, unstimulated), control (CHO-conditioned media), 0.5 µg/ml rAIBP, or 7PA2-conditioned media at the indicated concentrations of Aβ for 48 hours (n=7/group).

(**B**) In reference to Figures 1C and 1D, image of cells subjected to proximity ligation assay (PLA) in which primary antibodies were omitted. Scale bar, 20 µm.

(**C**) Validation of *Apoa1bp* knockout in mouse brain lysates by RT-qPCR: *Apoa1bp* mRNA expression (n=3 per group).

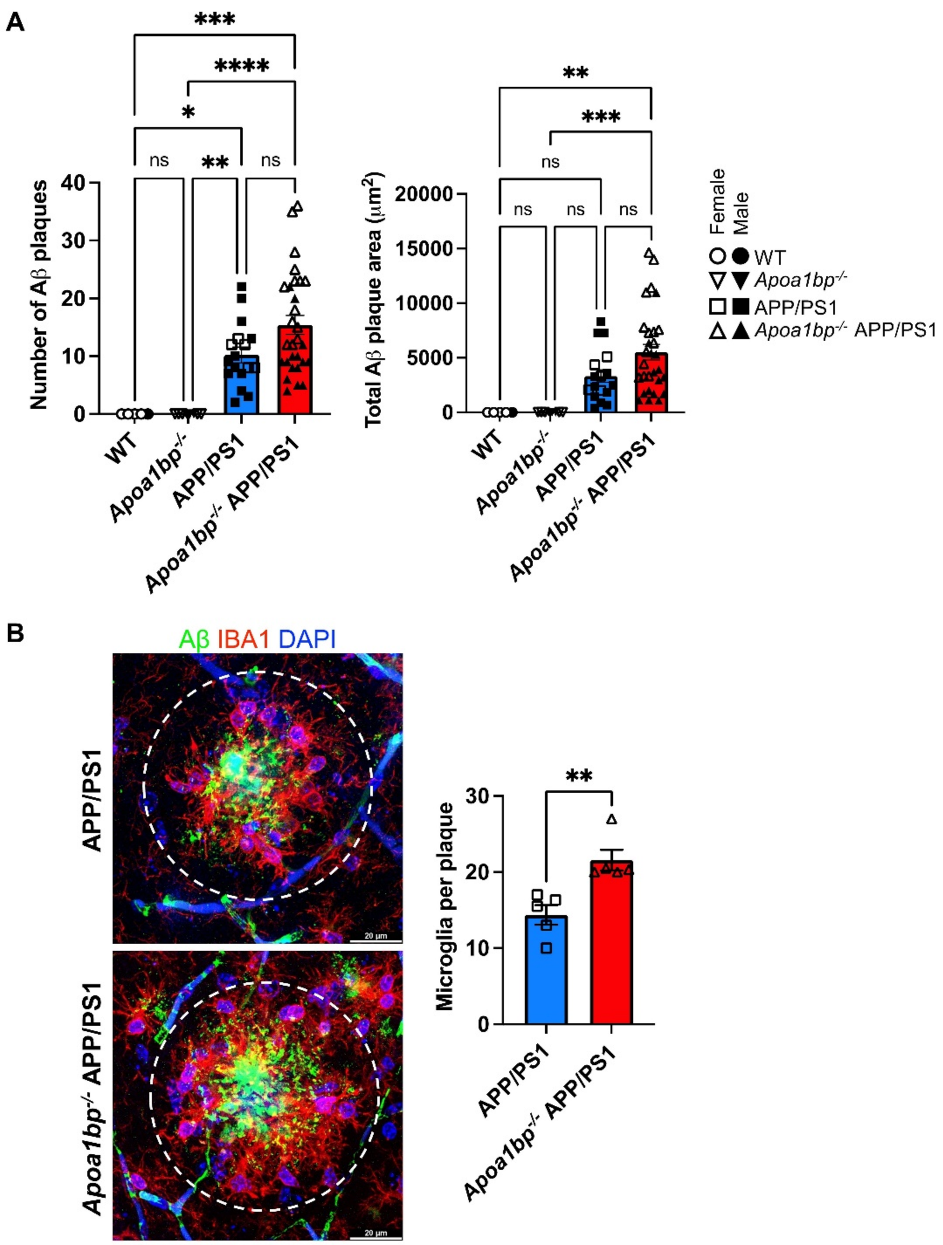

Figure S2. Supplemental data for Figure 7.

(**A**) The number of plaques and the total plaque area in the hippocampus of female and male mice (only females are shown in Figure 7). Data are from age-matched 6 months old male and female mice: WT (*n*=5), *Apoa1bp*$^{-/-}$ (*n*=8), APP/PS1 (*n*=17), and *Apoa1bp*$^{-/-}$ APP/PS1 (*n*=29). Open symbols represent females, and closed symbols represent males. Mean±SEM. One-way ANOVA with Tukey's multiple comparison test.

(**B**) Representative higher magnification image of hippocampus: 82E1 (Aβ; green), IBA1 (microglia; red), and DAPI (nuclei; blue). Scale bar, 20 μm.

The numbers of Aβ plaques-associated microglia (inside the circle) were counted in 2-3 different plaque-associated areas in the brain of 5 female mice per group. Mean±SEM. Unpaired t-test.

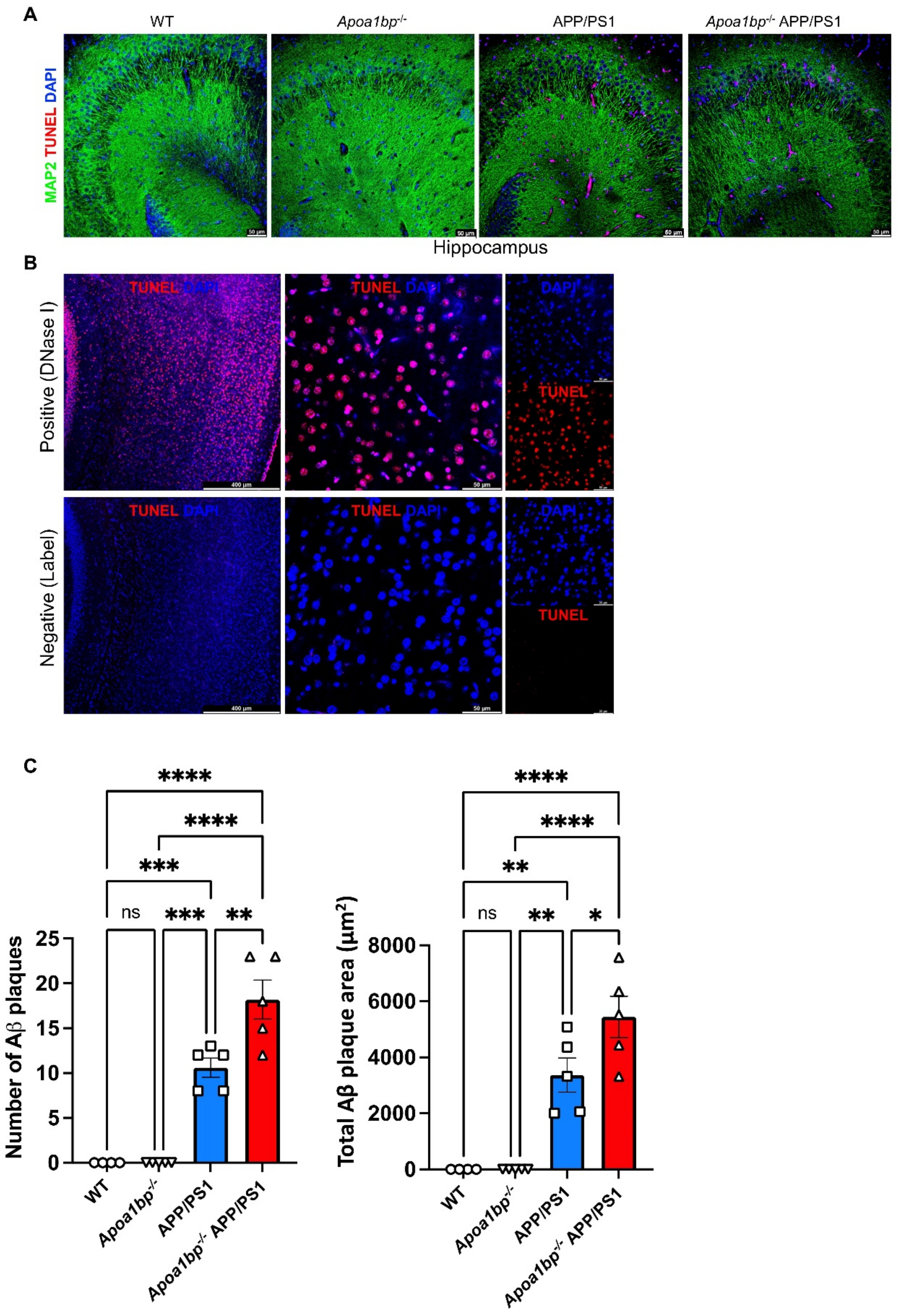

Figure S3. Controls and supplemental data for Figure 8.
(**A**) Lower magnification (wider field) images of TUNEL (red), MAP2 (green), and DAPI (blue) staining in hippocampus from female mice. Scale bar, 50 µm.
(**B**) Positive control (DNase I) and negative control (Label solution only) of *In Situ* Cell Death Detection TMR red Kit (TUNEL) in female WT mouse brain. Scale bars, 400 µm (left) and 50 µm (center and right).
(**C**) The number of plaques and the total plaque area in the hippocampus of mice used for experiment shown in Figure 8 (TUNEL and MAP2). Mean±SEM. One-way ANOVA with Tukey's multiple comparison test.

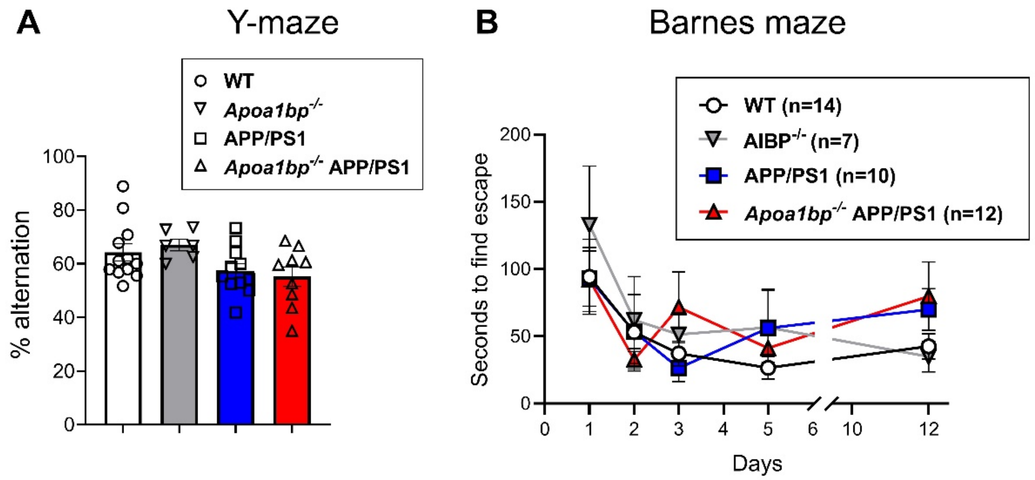

Figure S4. Behavioral data for WT, *Apoa1bp*$^{-/-}$, APP/PS1 and *Apoa1bp*$^{-/-}$ APP/PS1 mice.
Mice were tested at 9 months of age with Y-maze (**A**) and at 10 months of age with Barnes maze (**B**). Combined data for males and females. Mean±SEM; one (**A**) and two (**B**) way ANOVA with Tukey's multiple comparisons test; numbers of animals as presented on the graph. No significant differences for combined male and female (shown on the graphs) nor for each sex analyzed separately.

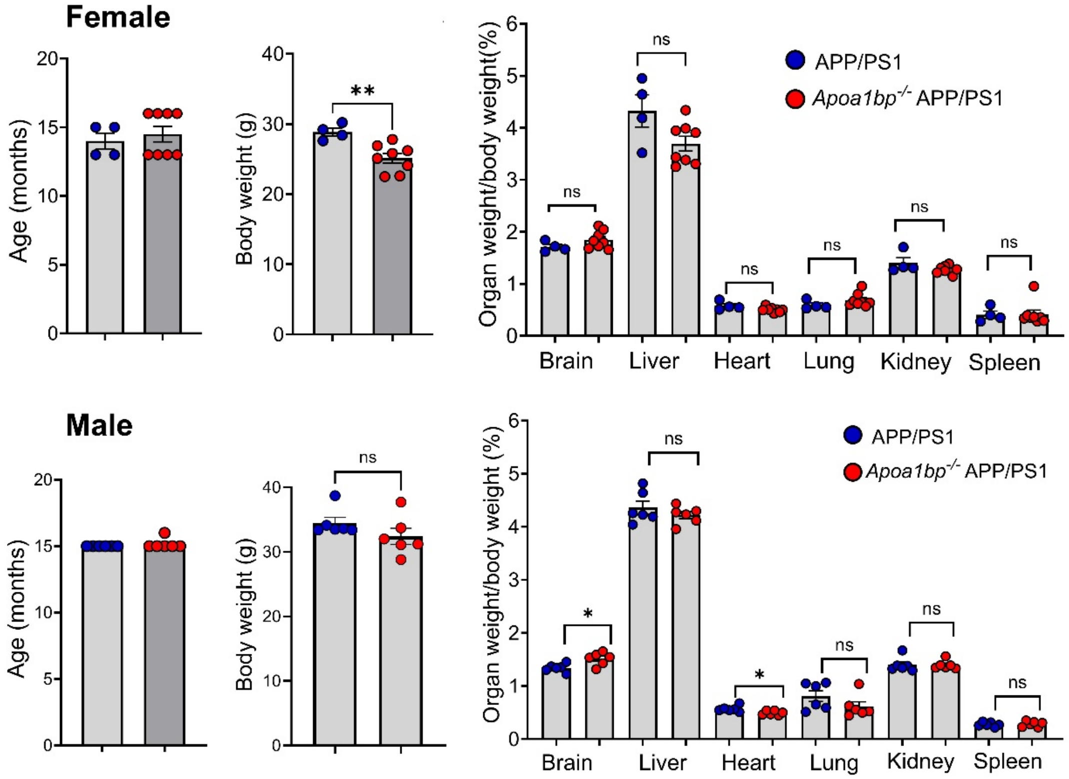

Figure S5. Related to Figure 8C: Body weight and organ weight in aged *Apoa1bp*$^{-/-}$ APP/PS1 and APP/PS1 mice.
Age distribution, body weight and internal organ weight normalized to body weight in APP/PS1 and *Apoa1bp*$^{-/-}$ APP/PS1 mice. Mean±SEM; unpaired, two-tailed t-test.
